# Current status of tertiary debulking surgery and prognosis after secondary debulking surgery for recurrent Müllerian epithelial cancer in Japan: a retrospective analysis of 164 patients (KCOG-G1402)

**DOI:** 10.1186/s12957-017-1200-x

**Published:** 2017-07-17

**Authors:** Tomoko Hirakawa, Takeo Minaguchi, Yoshio Itani, Yuka Kasamatsu, Saki Murase, Shoko Sakurada, Hiroaki Nagano, Kazuhiro Takehara, Tomohiko Tsuruta, Atsushi Arakawa, Kouichiro Kawano, Hiroshi Tsubamoto, Takashi Ushiwaka, Taisuke Mori, Kana Iwai, Motoaki Saito, Hiroyuki Morisawa, Fumitaka Saito, Kenta Yoshida, Masanori Kaneuchi, Hiroki Sato, Kimihiko Ito, Kaei Nasu

**Affiliations:** 1 Kansai Clinical Oncology Group (KCOG), 5-30 Tennoji, Osaka, 543-8555 Japan; 2 Department of Obstetrics and Gynecology, Faculty of Medicine, Oita University, 1-1 Idaigaoka, Hasama-machi, Yufu-shi, Oita 879-5593 Japan; 3 Department of Obstetrics and Gynecology, Faculty of Medicine, University of Tsukuba, Tsukuba, Ibaraki 305-8576 Japan; 4 Department of Obstetrics and Gynecology, Nara Prefecture General Medical Center, Nara, 631-0846 Japan; 5 Division of Gynecology, Shizuoka Cancer Center Hospital, Suntou, Shizuoka 411-8777 Japan; 6 Department of Obstetrics and Gynecology, Gifu University, Gifu, 501-1194 Japan; 7 Department of Obstetrics and Gynecology, Tohoku University School of Medicine, Sendai, Miyagi 980-8574 Japan; 8 Department of Obstetrics and Gynecology, Tokyo Women׳s Medical University Medical Center East, Arakawa, Tokyo, 116-8567 Japan; 9 Department of Gynecologic Oncology, Shikoku Cancer Center, Matsuyama, Ehime 791-0280 Japan; 10 Department of Obstetrics and Gynecology, Kansai Rosai Hospital, Amagasaki, Hyogo 660-8511 Japan; 11 Department of Obstetrics and Gynecology, Nagoya City University Graduate School of Medical Sciences, Nagoya, Aichi 467-8602 Japan; 12 Department of Obstetrics and Gynecology, Kurume University School of Medicine, Kurume, Fukuoka 830-0011 Japan; 13 Department of Obstetrics and Gynecology, Hyogo College of Medicine, Nishinomiya, Hyogo 663-8501 Japan; 14 Department of Obstetrics and Gynecology, Kochi Medical School, Nankoku, Kochi 783-8505 Japan; 15 Department of Obstetrics and Gynecology, Kyoto Prefectural University of Medicine, Kyoto, 602-8566 Japan; 16 Department of Obstetrics and Gynecology, Nara Medical University, Kashihara, Nara 634-8521 Japan; 17 Department of Obstetrics and Gynecology, Jikei University School of Medicine, Minato-ku, Tokyo, 105-8471 Japan; 18 Department of Obstetrics and Gynecology, Jichi Medical University, Shimotsuke, Tochigi 329-0498 Japan; 19 Department of Obstetrics and Gynecology, Faculty of Life Science, Kumamoto University, Kumamoto, 860-8556 Japan; 20 Department of Obstetrics and Gynecology, Mie University School of Medicine, Tsu, Mie 514-8507 Japan; 21 Department of Obstetrics and Gynecology, Nagasaki University, Nagasaki, 852-8501 Japan

**Keywords:** Müllerian epithelial cancer, Recurrence, Secondary debulking surgery, Tertiary debulking surgery, Quaternary debulking surgery

## Abstract

**Background:**

This study aimed to evaluate the current status of secondary debulking surgery (SDS) and tertiary debulking surgery (TDS; performed for recurrence after SDS) and to assess the overall survival after recurrence of Müllerian epithelial cancer in Japan. We also evaluated the data of patients who underwent a fourth debulking surgery (i.e., quaternary debulking surgery (QDS)).

**Methods:**

We conducted a retrospective study of 164 patients with recurrent Müllerian epithelial cancers (i.e., ovarian, tubal, and peritoneal cancers). The SDS was performed between January 2000 and September 2014 in 20 Japanese hospitals. Clinicopathological data were collected and analyzed.

**Results:**

Of the 164 patients, 66 patients did not have a recurrence or died after SDS. Ninety-eight patients had a recurrence after SDS. Forty-three of the 98 patients underwent TDS; 55 of the 98 patients did not undergo TDS and were classified into the non-TDS group. The overall survival (OS) after SDS was significantly better in the TDS group than in the non-TDS group. The median OS after SDS was 123 and 42 months in the TDS group and non-TDS group, respectively. Of the 43 patients who received TDS, 11 patients were further treated with QDS. The median OS after SDS was 123 months for patients who underwent QDS.

**Conclusions:**

This multicenter study on the prognosis of post-SDS is apparently the first report on QDS in Japan. Patients undergoing TDS have a good prognosis, compared to patients in the non-TDS group. Novel drugs are being evaluated; however, debulking surgery remains a necessary treatment for recurrence.

## Background

The treatment of recurrent ovarian cancer consists mainly of chemotherapy. However, secondary debulking surgery (SDS; also known as secondary cytoreductive surgery) is an effective option if the patients are selected carefully [1–4]. There are very few reports on Japanese patients treated with SDS, although there is one previous report on 44 Japanese patients [5]. Secondary debulking surgery seems to be effective if complete resection has been achieved. The AGO DESKTOP OVAR Trial (DESKTOP I trial) showed that only complete surgery was associated with prolonged survival in patients with recurrent ovarian cancer [6]. Moreover, the trial presented a predictive score for the resectability of recurrent ovarian cancer. This score model was based on the performance status, the presence of ascites, and the outcome of the primary surgery [6, 7]. The DESKTOP II trial was subsequently the first prospective multicenter trial that successfully validated a clinical score that could be used to predict complete resection [8].

Surgery after SDS (i.e., tertiary debulking surgery (TDS)) is performed in highly selected cases and is rarely reported [9–13]. Moreover, reports of a fourth debulking surgery (i.e., quaternary debulking surgery (QDS)) are even rarer [14–17]. If the efficacy of SDS can be proven, the effectiveness of TDS or additional debulking surgeries should be studied.

With this in mind, the aims of this study were to retrospectively analyze the data of patients with recurrent Müllerian epithelial cancers (i.e., ovarian, tubal, and peritoneal cancers) and to assess the prognostic factors and the current status of recurrence after SDS in Japan.

## Methods

We designed a multicenter retrospective study, which included 164 patients with recurrent Müllerian epithelial cancer who were enrolled from 20 hospitals in Japan. The Institutional Review Boards of each hospital approved this study. The patient selection criteria were as follows: a complete response was achieved by the primary treatment, patients were histologically diagnosed with Müllerian epithelial cancers (i.e., ovarian, tubal, and peritoneal cancers), and SDS was performed between January 2000 and September 2014. The performance status of the patients was estimated to be 0 or 1, based on the Eastern Cooperative Oncology Group Performance Status (ECOG-PS) criteria. The exclusion criteria were as follows: a history of other cancer, patients who did not achieve complete response by SDS or subsequent chemotherapy, and patients who underwent palliative surgery at first recurrence (therefore, it was not regarded as SDS). The collected data about the initial treatment included age at the initial diagnosis, clinical and pathological stage (i.e., International Federation of Gynecology and Obstetrics (FIGO) stage), histology, serum cancer antigen-125 (CA125) level, and presence of residual tumor (0, <10, or ≥10 mm) at the primary debulking surgery (PDS). The data collected at recurrence included age, platinum-free interval, serum CA125 level, presence of peritonitis carcinomatosa, and presence of residual tumors at SDS (0, <10, or ≥10 mm). The collected data at the TDS (i.e., recurrence after SDS) were age, serum CA125 level, presence of peritonitis carcinomatosa, site of recurrence, and number of recurrence. Survival time after SDS and data regarding TDS and QDS were also collected. The absence of residual tumor (0 mm) was defined as a complete surgery. The TDS group consisted of patients who underwent debulking surgery for recurrence after SDS. Patients undergoing a fourth or fifth debulking surgery were also included in the TDS group. The non-TDS group consisted of patients who did not undergo a TDS; these patients had not undergone surgery for recurrence after the SDS.

The primary objective was to compare the overall survival (OS) in the TDS group with that in the non-TDS group. The OS was the interval between the date of the SDS and death. Most studies define OS as the survival time from the initial diagnosis. However, in this study, all patients underwent a SDS. The time between the primary treatment and recurrence ranged widely among them; therefore, the definition of OS as the survival time between the date of SDS and death was appropriate.

The secondary objective was to identify the characteristics of the TDS group and the non-TDS group. The platinum-free interval was the interval between the date of the last infusion of platinum for the primary treatment and the date of recurrence. Platinum resistance was defined as recurrence within 6 months after the last platinum treatment. Platinum partial sensitivity was defined as recurrence between 6 and 12 months after platinum treatment. Platinum sensitivity was defined as recurrence after 12 months.

The differences in the background characteristics of the patients were analyzed using the chi-square test. The survival data were analyzed using the Kaplan–Meier method and log-rank test. For all analyses, Statistical Package for Social Science software (IBM SPSS Statistics 24; IBM, Armonk, NY) was used, and *P* < 0.05 was considered statistically significant.

## Results

**Fig. 1:**
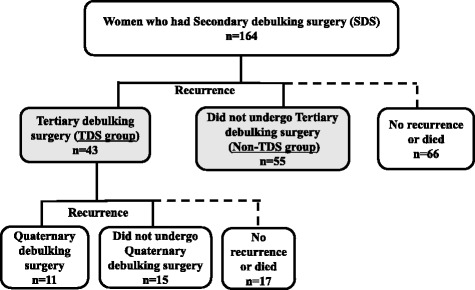
The inclusion criteria of patients in this study

Figure 1 shows the inclusion criteria for data collection in this study. One hundred sixty-four patients underwent SDS in 20 hospitals. Sixty-six patients did not have a recurrence or died after SDS. Ninety-eight patients had a recurrence after SDS. Forty-three of the 98 patients were classified into the TDS group. Tertiary debulking surgery was performed in 15 hospitals. Among the TDS group, 11 (25.6%) patients underwent QDS, 15 (34.9%) patients did not undergo QDS, and 17 (39.5%) patients did not have a recurrence or died after TDS.

Fifty-five of the 98 patients did not undergo TDS when they had a recurrence after SDS, and they were classified into the non-TDS group. The non-TDS patients were treated with chemotherapy or the best supportive care.

Table 1 shows the main characteristics of the patients in two groups at the time of initial diagnosis, SDS, and recurrence after SDS. The median ages of the patients at initial diagnosis were 56.9 years (range, 35–74 years) and 54.2 (range, 27–77 years) in the TDS and non-TDS groups, respectively. Forty-two (97.7%) patients in the TDS group and 51 (92.7%) patients in the non-TDS group were diagnosed with ovarian cancer. No cases of tubal cancer were included in the TDS group. There were only two (3.6%) cases of tubal cancer in the non-TDS group. One (2.3%) patient in the TDS group and two (3.6%) patients in the non-TDS group had peritoneal cancer. Serous cystadenocarcinoma was the most common histological type (22 [51.2%] cases in the TDS group and 36 [65.5%] cases in the non-TDS group). Complete surgery at PDS was performed in 34 (79.1%) patients in the TDS group and 33 (60.0%) patients in the non-TDS group. Complete surgery at SDS was performed in 38 (88.4%) patients in the TDS group and 44 (80.0%) patients in the non-TDS group.

**Table 1:** Clinical characteristics of the tertiary debulking surgery (TDS) group and non-TDS group

Characteristics	TDS (*n* = 43)	Non-TDS (*n* = 55)	*P* value
Primary			
Age, median (range), years	56.9 (35–74)	54.2 (27–77)	
<60	24 (55.8%)	35 (63.6%)	0.432
≧60	19 (44.2%)	20 (36.4%)	
Cancer			
Ovary	42 (97.7%)	51 (92.7%)	0.415
Fallopian tube	0 (0%)	2 (3.6%)	
Peritoneum	1 (2.3%)	2 (3.6%)	
FIGO stage			
I, II	10 (23.3%)	13 (23.6%)	0.965
III, IV	33 (76.7%)	42 (76.4%)	
Histology			
Serous	22 (51.2%)	36 (65.5%)	0.333
Endometrioid	6 (14.0%)	4 (7.3%)	
Clear cell	10 (23.3%)	6 (10.9%)	
Mucinous	2 (4.7%)	3 (5.5%)	
Others	3 (7.0%)	6 (10.9%)	
CA 125 (U/ml)			
< 100	10 (23.3%)	7 (12.7%)	0.086
≧100	21 (48.8%)	38 (69.1%)	
Missing	12 (27.9%)	10 (18.2%)	
Residual disease at PDS, mm			
0	34 (79.1%)	33 (60.0%)	0.035
0-10	8 (18.6%)	11 (20%)	
≧10	1 (2.3%)	10 (18.2%)	
Missing	0 (0%)	1 (1.8%)	
Recurrence at SDS			
Age, median (range), years	59.5 (40–76)	56.8 (32–79)	
<60	20 (46.5%)	30 (54.5%)	0.430
≧60	23 (53.5%)	25 (45.5%)	
Platinum-free interval			
<6 months	4 (9.3%)	3 (5.5%)	0.09
6–12 months	13 (30.2%)	9 (16.4%)	
≧12 months	22 (51.2%)	42 (76.4%)	
Missing	4 (9.3%)	1 (1.8%)	
CA 125 (U/ml)			
<100	32 (74.4%)	37 (67.3%)	0.410
≧100	8 (18.6%)	14 (25.5%)	
Missing	3 (7.0%)	4 (7.3%)	
Peritonitis carcinomatosa			
No	39 (90.7%)	48 (87.3%)	0.920
Yes	3 (7.0%)	4 (7.3%)	
Missing	1 (2.3%)	3 (5.5%)	
Residual disease at SDS, mm			
0	38 (88.4%)	44 (80.0%)	0.092
0–10	1 (2.3%)	8 (14.5%)	
≧10	2 (4.7%)	1 (1.8%)	
Missing	2 (4.7%)	2 (3.6%)	
Recurrence at TDS			
Age, median (range), years	61.5 (40–78)	58.6 (33–80)	
<60	17 (39.5%)	28 (50.9%)	0.262
≧60	26 (60.5%)	27 (49.1%)	
CA 125 (U/ml)			
<100	31 (72.1%)	30 (54.5%)	0.040
≧100	7 (16.3%)	19 (34.5%)	
Missing	5 (11.6%)	6 (10.9%)	
Peritonitis carcinomatosa			
No	40 (93.0%)	46 (83.6%)	0.042
Yes	0 (0%)	5 (9.1%)	
Missing	3 (7.0%)	4 (7.3%)	
Sites of recurrence			
Peritoneal	28 (65.1%)	48 (87.3%)	0.009
Extra-peritoneal	15 (34.9%)	7 (12.7%)	
Number of recurrence			
Single	24 (55.8%)	17 (30.9%)	0.013
Multiple	19 (44.2%)	38 (69.1%)	

The median ages at TDS recurrence (i.e., recurrence after SDS) were 61.5 years (range, 40–78 years) and 58.6 years (range, 33–80 years) in the TDS and non-TDS groups, respectively. The CA125 levels at TDS recurrence (i.e., recurrence after SDS) significantly differed between the two groups (*P* = 0.040). Peritonitis carcinomatosa was significantly different in the non-TDS patients (*P* = 0.042). With regard to the sites of recurrence, more patients showed recurrence in the peritoneal cavity in the non-TDS group than in the TDS group (*P* = 0.009). In the TDS and non-TDS groups, peritoneal recurrence occurred in 28 (65.1%) patients and 48 (87.3%) patients, respectively, while extra-peritoneal recurrence occurred in 15 (34.9%) patients and seven (12.7%) patients, respectively. Among cases of extra-peritoneal recurrence in the TDS group, five (33.3%) cases occurred in the lung; five (33.3%) cases occurred in the brain; four (26.7%) cases occurred in the inguinal lymph nodes; and one (6.7%) case occurred in the axilla. In the non-TDS group, the extra-peritoneal recurrence site was the lung in three (42.9%) cases, the mediastinal space in three (42.9%) cases, and the bone in one (14.3%) case. With regard to the number of recurrence sites, more patients presented with single-site recurrence in the TDS group than in the non-TDS group (*P* = 0.013). Solitary recurrence occurred in 24 (55.8%) TDS patients and 17 (30.9%) non-TDS patients, and multiple recurrences occurred in 19 (44.2%) TDS patients and 38 (69.1%) non-TDS patients.

**Fig. 2:**
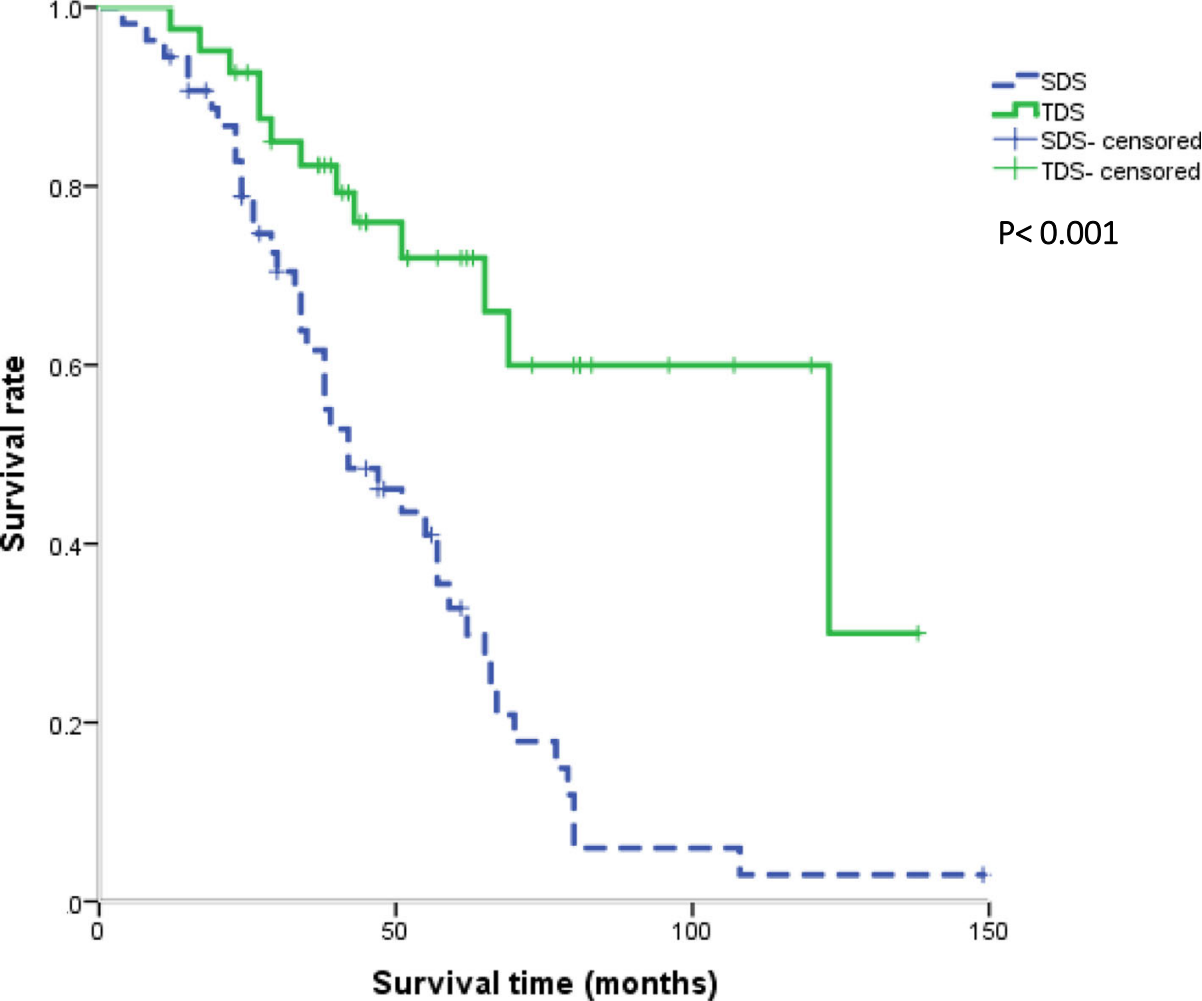
Kaplan–Meier plot of survival rates after SDS. The survival rate was significantly better in the TDS group than in the non-TDS group (*P* < 0.001). The median OS after SDS was 123 months in the TDS group and 42 months in the non-TDS group. *SDS* secondary debulking surgery, *TDS* tertiary debulking surgery, *OS* overall survival

Among the 164 patients included in this study, 43 patients underwent TDS. The OS after SDS was significantly better in the TDS group, compared to the non-TDS group (*P* = 0.001; Fig. 2). The median OS after SDS was 123 months and 42 months in the TDS group and non-TDS group, respectively.

Our study included 11 patients who underwent QDS (Table 2). Moreover, two of the 11 QDS patients underwent a fifth debulking surgery. Eight of the 11 patients were alive at the conclusion of the study. The median age of the 11 patients at QDS was 57 years (range, 52–65 years), and the median OS after SDS was 123 months (range, 43–138 months; Table 2).

**Table 2:** Patients characteristics at the time of quaternary debulking surgery (QDS)

Author	Year	Number	FIGO stage (*n*)	Age^a^	Residual disease (*n*)	Median CA125 (U/ml)	Chemotherapy (*n*)	Median OS (m)^b^
Shih et al.	2010	15	I (1)	54.1	0 mm (10)	NA	Yes (9)	34.8, 10.1 (from TDS)^c^
			II (2)		>0 mm (5)		No (6)	
			III (9)					
			IV (1)					
Fotopoulou et al.	2013	49	I (6)	57	0 mm (16)	736	Yes (18)	23.05 (from QDS)
			II (6)		>0 mm (33)		No (31)	
			III (33)					
			IV (1)					
Bacarubasa et al.	2015	20	I (4)	54.3	0 mm (7)	NA	NA	16 (from QDS)
			II (5)		>0 mm (13)			
			III (11)					
			IV (0)					
Hirakawa et al.	2017	11	I (0)	57	0 mm (7)	11.2 (6 cases)	Yes (4)	123 (from SDS)
			II (1)		>0 mm (2)		No (7)	
			III (7)		Missing (2)			
			IV (3)					

*QDS* quaternary debulking surgery, *NA* data were not available

^a^The values are the median age at QDS

^b^The values are the median overall survival (OS)

^c^For residual disease ≤10 mm, the median OS was 34.8 months; for residual disease >10 mm, the median OS was 10.1 months

## Discussion

This study aimed to clarify the current status of SDS and TDS in Japan because few patients have undergone SDS or TDS in Japan and the current status of SDS and TDS is unknown. We found no significant difference in the characteristics between the TDS and non-TDS groups at the primary treatment and at the first recurrence, except for residual disease at PDS. However, a significant difference was noted between the TDS and non-TDS groups in the survival time from the SDS (*P* < 0.001, Fig. 2). It has been previously demonstrated that SDS shows an advantage in terms of longer OS for highly selected patients [18, 19]; however, the predictors of survival in these patients remain unclear.

It has been reported that ascites (<500 ml) is a prognostic factor and predictor of complete surgery [6, 7]. In this study, it was difficult to collect data concerning ascites because amount of ascites was not measured in most patients. Therefore, we evaluated ascites by using the presence of peritonitis carcinomatosa. In the present study, only three (7.0%) patients and four (7.3%) patients relapsed with carcinomatous peritonitis in the TDS and non-TDS groups at SDS, respectively. It is possible that the patients with ascites were treated largely by chemotherapy instead of SDS, although this study has no data on the non-SDS patients (i.e., patients who did not receive SDS at first recurrence). At the time of TDS (i.e., recurrence after SDS), more patients in the non-TDS group had a recurrence in the peritoneal cavity, compared to the TDS group (*P* = 0.009). Moreover, more patients showed single-site recurrence in the TDS group than in the non-TDS group (*P* = 0.013). Many cases of peritoneal cavity recurrence involved wide peritoneal dissemination. These patients may have selected chemotherapy instead of TDS.

In the TDS group, 11 patients underwent QDS and two of these 11 patients underwent a fifth debulking surgery. Ten (90.9%) of the 11 patients with FIGO stage III and IV cancers were in the QDS group. This study included higher ratio of advanced stage cancer patients, compared to other reports on patients with QDS (Table 2). Based on these results, we believe that this is a valuable study to clarify the current status of TDS or additional debulking surgeries.

There are several limitations of this study. One of two main limitations is the retrospective nature of the study. The second main limitation is the small sample size per year and the small number of institutions. Among the 164 patients over 14 years, only approximately 12 patients underwent SDS per year across 20 institutions. We assumed that very few Japanese patients were treated with SDS. Therefore, we collected data from many hospitals in Japan. Despite the smaller sample size per institution, this study provides valuable data concerning TDS and QDS.

A third limitation is the potential for selection bias, such as similar tumor sizes and number of relapse sites. This bias might influence the presence of residual tumor after debulking surgery and/or the survival time. In future studies, selection bias in evaluating the efficacy of SDS and TDS needs to be overcome. We could not rule out the possibility that only highly selected patients were operated on in the present study; nevertheless, this is a valuable study on the current status of SDS and TDS. To the best of our knowledge, this is the first report on TDS and QDS in Japan.

## Conclusions

In conclusion, several novel agents such as monoclonal antibodies are being evaluated, although debulking surgery remains an important treatment option for multiple relapses. The present study demonstrated that, at second recurrence, patients who were treated with TDS showed a favorable OS, compared to patients treated with chemotherapy or best supportive care. Further reports of patients treated with SDS or additional debulking surgery need to be accumulated to evaluate treatment results and confirm our findings.

## Abbreviations

OS: Overall survival
QDS: Quaternary debulking surgery
SDS: Secondary debulking surgery
TDS: Tertiary debulking surgery
